# The Influence of Post-Treatment on Micropore Evolution and Mechanical Performance in AlSi10Mg Alloy Manufactured by Laser Powder Bed Fusion

**DOI:** 10.3390/ma17174319

**Published:** 2024-08-30

**Authors:** Qing Pu, Jinbiao Qian, Yingwei Zhang, Shangjing Yang, Hongshou Huang, Qi Chao, Guohua Fan

**Affiliations:** 1 Key Laboratory for Light-Weight Materials, Nanjing Tech University, Nanjing 210037, China; pupuxx0102@163.com (Q.P.); 202161203233@njtech.edu.cn (J.Q.); 2 AVIC Shenyang Aircraft Industrial (Group) Co., Ltd., Shenyang 110850, China; zhangyw004@avic.com; 3 Materials Academy, Jiangsu Industrial Technology Research Institute (JITRI), Suzhou 215000, China; yangshangjing.2007@163.com; 4 Weikuang Technology (Suzhou) Co., Ltd., Suzhou 215000, China; 5 Future Industries Institute (FII), University of South Australia, Mawson Lakes, SA 5095, Australia; hongshou.huang@mymail.unisa.edu.au

**Keywords:** laser additive manufacturing, aluminum alloys, laser powder bed fusion, porosity, heat treatment, mechanical property

## Abstract

Gas-induced porosity is almost inevitable in additively manufactured aluminum alloys due to the evaporation of low-melting point elements (e.g., Al, Mg, and Zn) and the encapsulation of gases (e.g., hydrogen) during the multiple-phase reaction in the melt pool. These micropores are highly unstable during post-heat treatment at elevated temperatures and greatly affect mechanical properties and service reliability. In this study, the AlSi10Mg samples prepared by LPBF were subjected to solution heat treatment at 560 °C for 0.5 and 2 h, followed by artificial aging at 160 °C, 180 °C and 200 °C, respectively. The defect tolerance of gas porosity and associated damage mechanisms in the as-built and heat treated AlSi10Mg alloy were elucidated using optical, scanning electron microscopic analysis, X-ray micro computed tomography (XCT) and room temperature tensile testing. The results showed the defect tolerance of AlSi10Mg alloy prepared by LPBF was significantly reduced by the artificial aging treatment due to the precipitation of Mg-Si phases. Fracture analysis showed that the cooperation of fine precipitates and coarsened micropores assists nucleation and propagation of microcracks sites due to stress concentration upon tensile deformation and reduces the tensile elongation at break.

## 1. Introduction

Laser powder bed fusion (LPBF) is a precise metal additive manufacturing technology widely employed for producing complex metal components in aerospace, automotive, medical, and other industries [1,2,3,4,5]. Despite its advantages, LPBF-processed metal parts often contain defects such as porosity, which significantly affect their mechanical, corrosion, and other properties [6,7]. The primary porosity defects in LPBF-processed metals include lack of fusion (LOF), keyholes, and gas pores. LOF defects typically form when the energy density applied to the melt pool is insufficient to fully fuse the metal powders. Keyhole defects occur when excessive laser power causes slight vaporization of metals, creating near-spherical pores in the fused layers [8,9,10,11,12]. For metals with good printability, optimizing processing parameters can reduce these defects to a quite low level (e.g., mean size ≤ 50 μm and total volume fraction ≤ 0.05%) where their negative impacts on material properties are minimal by optimizing the processing parameters [13,14]. However, gas porosity is common in LPBF-processed light metals such as aluminum (Al) alloys, due to the evaporation of low-melting point elements (e.g., Al, Mg, Zn) and the encapsulation of gases like hydrogen in the melt pool [15]. Since Al and its major alloying elements have a high affinity for hydrogen, hydrogen can be significantly dissolved during the heating and solidification processes of aluminum alloys, making it difficult to control in LPBF-treated Al alloys [16,17,18]. They absorb hydrogen during production, storage, use, and recycling. Li et al. [17] found that drying Al alloy powder at 100 °C for 1 h prior to LPBF use increased the build’s relative density to over 99%. However, it is difficult to eliminate hydrogen-induced porosity, even after drying Al powder at 200 °C [19]. In practice, most powder drying operations are carried out below 200 °C for Al alloys as long-term drying at higher temperatures is not safe and can cause agglomeration or bonding between powders. Additionally, gas-induced porosity is unstable upon high-temperature post-treatment and exhibits remarkable growth and aggregation at a holding temperature of over 450 °C [20]. Strumza et al. [21] investigated the evolution of thermally induced porosity in the AlSi10Mg produced by LPBF using thermal programmed desorption mass spectrometry analysis. They found the thermally induced expansion of trapped hydrogen is the main source for the pressure increase in pores that leads to permanent expansion upon heating up to 550 °C.

For many LPBF-processed aluminum alloys, post-heat treatments are essential to further improve their strength (e.g., AA2219 alloy) and ductility (e.g., AlSi10Mg alloy) [22,23,24]. AlSi10Mg alloy is one of the most commonly used aluminum alloys for LPBF due to its good weldability, weight-to-strength ratio, and thermal conductivity [8,25]. The LPBF-processed AlSi10Mg alloy is usually subjected to solution treatment at 510–540 °C, followed by artificial aging at 120–190 °C for 1-4 h to improve its mechanical properties [26]. Zhu et al. [27] explored the effects of T5 (artificial aging at 170 °C for 2 h) and T6 (solution treatment at 520 °C for 2 h followed by artificial aging at 170 °C for 8 h) treatments on the microstructure and mechanical properties of LPBF-processed AlSi10Mg alloy. They found that T5 treatment increased both yield strength (YS) and ultimate tensile strength (UTS) through aging hardening but reduced ductility. In contrast, T6 treatment provided a better combination of ductility and YS due to the synergistic effects of Al-Si network spheroidization and needle-like β’’ phase precipitation strengthening.

While most research has focused on the impact of microstructural evolution during heat treatment on the mechanical properties of additive-manufactured Al alloys [28,29,30], studies addressing the evolution and critical tolerance of micropores are limited. Hastie et al. [31] investigated the influence of processing parameters and post-heat treatment on micropore formation and evolution within LPBF-processed AlSi10Mg alloy using X-ray microcomputed tomography (XμCT) but did not link these findings to mechanical properties. Generally, densifying the as-built material through parameter optimization during LPBF or post-heat treatment, such as hot isostatic pressing (HIPing), can reduce porosity and improve mechanical performance. However, Giovagnoli et al. [32] found that solution treatment at 520 °C for 2 h reduced the relative density from 99.8% in as-built state to 97.8% due to the growth of pre-existing pores. Nonetheless, the defect tolerance and damage mechanism of LPBF-manufactured AlSi10Mg alloy after T6 treatment remain unclear.

This study aims to tailor the gas porosity characteristics, including size and proportion, in LPBF-processed AlSi10Mg alloy by varying the solid solution time, and to investigate their impact on thermal stability and critical tolerance of porosity in the presence of a considerable number of precipitates (e.g., Mg_2_Si [33]). A combination of optical, electron microscopes, and XCT technology was employed to examine the effects of post-treatments on micropore evolution and the mechanical performance of LPBF-processed AlSi10Mg alloy.

## 2. Materials and Experimental Method

**Figure 1:**
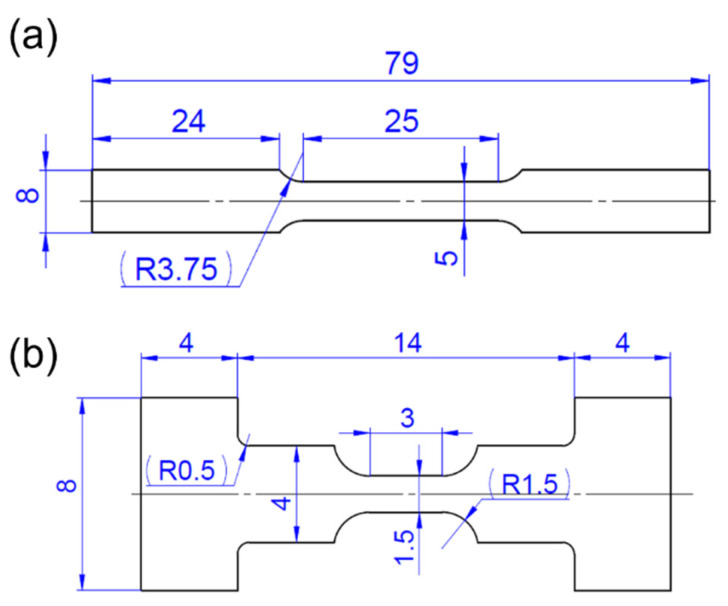
Schematic diagram of plate samples for conventional tensile test (**a**) and for interrupted in-situ tensile test with XCT (**b**). The samples with a thickness of ~1.5 mm were machined along the build direction. Dimensions shown in millimeters.

The AlSi10Mg powder, with a particle size range of 15–53 μm, used in this study was supplied by Xi’an Bright Laser Technology Co., Ltd. (Xi’an, China). The chemical composition of the raw powder is 11.7% Si, 0.6% Mg, 0.3% Fe, and the balance Al (by weight). The AlSi10Mg powders were melted and deposited using a BLT-S210 (Bright Laser Technologies, Xi’an, China) LPBF system equipped with a 500 W IPG fiber laser with an 80 μm laser spot size. Rectangular plates with a size of 2 mm × 10 mm × 80 mm (X:Y:Z) were vertically deposited on an AA6061 substrate plate using a laser power of 375 W, a scanning speed of 1400 mm/s, a layer thickness of 30 μm, and a hatch distance of 100 μm. To study the thermal stability of micropores, the as-built plates were subjected to solution treatment at 560 °C for 0.5 and 2 h in a muffle furnace, followed by water quenching. The as-built and solution-treated samples were then aged at 160 °C, 180 °C, and 200 °C for durations of 1, 2, 4, and 8 h, respectively. Vickers hardness was measured using a Shimadzu HMV-G hardness tester(Shimadzu, Kyoto, Japan), with a test load of 0.5 kg and a load retention of 10 s. At least 5 measurements were conducted for each sample. The as-built and heat-treated plates were machined into tensile coupons of various sizes (as shown in Figure 1) using electric discharge machining (EDM). The tensile specimens with a gauge length of 25 mm (Figure 1a) were subjected to room-temperature quasi-static tensile testing at a true strain rate of 10^−3^/s using an Instron 5982 testing machine equipped with a non-contact video extensometer. The work hardening rate θ reflects a material’s capacity to produce and accumlate dislocatios for bearing higher load during plastic deformation and is determined by the equation of $\theta ={d\sigma }_{true}/{d\varepsilon }_{true}$, where $\sigma_{true}$ and $\varepsilon_{true}$ represent the corresponding true stress and true strain, respectively. Smaller tensile specimens with a gauge length of 3 mm (Figure 1b) were used for interrupted tensile testing at the same strain rate, employing a Weikuang XLAB-2000 (Weikuang Technology, Suzhou, China) in-situ X-ray computed tomography (XCT) instrument integrated with an optical extensometer.

To investigate the response of micropores during deformation, the tensile samples were scanned by XCT interrupted at different strains. The heat-treated and deformed samples were scanned using a spatial resolution of approximately 4.0 μm. The XCT data were reconstructed using Avizo 2023.1 software (Thermo Fisher Scientific, Waltham, MA, USA). Pores with a total volume of less than 15 μm^3^ were discarded, as they are unlikely to be properly detected by the XCT at the current resolution and their impact on tensile properties is minimal [34]. For each condition, at least three samples were tested, and the entire gauge length volume was scanned by XCT. The heat-treated and deformed samples were subjected to back-scattered electron (BSE) and electron backscattered diffraction (EBSD) analysis. The samples for microstructural characterization were prepared following the standard specimen preparation procedure, finished with 0.05 μm diameter oxide silicates (O-PS) [35]. Some specimens for optical microscope (OM, Carl Zeiss NTS GmbH, Oberkochen, Germany) and scanning electron microscope (SEM, Tescan MIRA(TESCAN, Brno, Czech Republic)) observation were further etched with Keller’s reagent (2.5% HNO_3_, 1% HF, 1.5% HCl, 95% H_2_O by volume). The EBSD data were acquired at 20 kV with a step size of 0.5 μm and processed using Oxford Aztec 2.1.2 software(Oxford Instruments, Oxford, UK).

## 3. Results and Discussion

### 3.1. Microstructure Evolution during Solution Treatment

**Figure 2:**
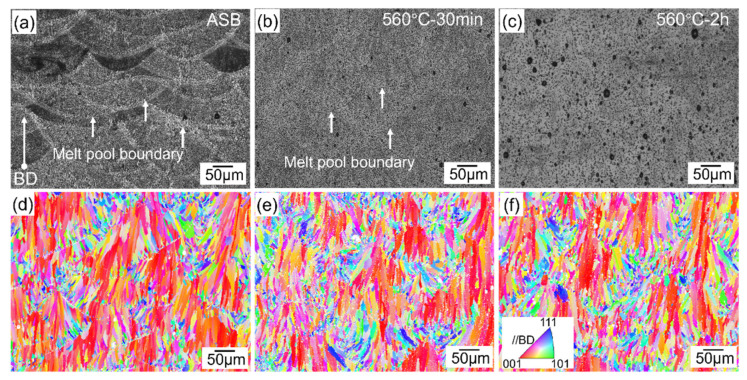
Micrographs of (**a**,**d**) as-built LPBF AlSi10Mg alloy, and solution treated samples at 560 °C for 30 min (**b**,**e**) and 2 h (**c**,**f**). (**a**–**c**) are optical micrographs, and (**d**–**f**) are EBSD inverse pole figure maps.

Figure 2a–c show the optical micrographs of the as-built and heat-treated samples. In the as-built (ASB) sample, a typical “fish scale”-like melt pool structure is observed (Figure 2a,d). This structural feature results from the axially symmetric Gaussian power density distribution of the laser beam, with the melt pool width and depth measuring approximately 142.3 μm and 76.3 μm, respectively. The melt pool mainly consists of columnar grains with (001) planes parallel to the build direction (Figure 2d). These columnar grains, averaging 22 ± 3.2 μm in size, grow epitaxially from the edge of the molten pool toward the centre, where more equiaxed, fine grains with diverse orientations are primarily located. The grain interiors are characterized by high Si content in the form of Al-Si eutectic networks, with widths ranging from 0.5 to 1 μm. In addition to the “fish scale”-like melt pools, columnar α-Al grains, and submicron cellular Al-Si networks [36], micropores ranging from 1.5 to 5 μm are also observed in the ASB microstructure. After solid solution heat treatment at 560 °C for 30 min and 2 h, the melt pool boundaries became less distinguishable due to the dissolution of Si into the α-Al matrix, and there was a remarkable increase in the size of micropores. Statistical analysis of the images (Figure 2a–c) shows that the volume fraction of micropores increased from 0.15% in the ASB state to 0.17% after heat treatment at 560 °C for 30 min, and to 0.65% after 2 h. This aligns with observations by Giovagnoli et al. [32]. The pores are randomly distributed throughout the sample, suggesting that microstructural changes might occur during the heat treatment. This porosity evolution is likely due to the increasing solubility and diffusion coefficient of hydrogen (H) in the aluminum alloy as the temperature rises. As the temperature increases, the hydrogen atoms become more mobile within the matrix [21]. This enhanced mobility allows for the growth or aggregation of micropores, as hydrogen atoms can diffuse towards the existing micropores or new nucleation sites. This leads to simultaneous increases in the mean size and volume fraction of micropores [37].

After solution treatment, no significant changes in grain size and orientation are observed compared to the as-built samples (Figure 2e,f). The characteristics of columnar grains inside the melt pools and fine equiaxed grains along the melt pool boundaries are mostly maintained. Similar observations have been reported by Zhu et al. [27]. This suggests that the as-built grain structure in LPBF-processed AlSi10Mg is rather stable even at the solid solution temperature [38].

**Figure 3:**
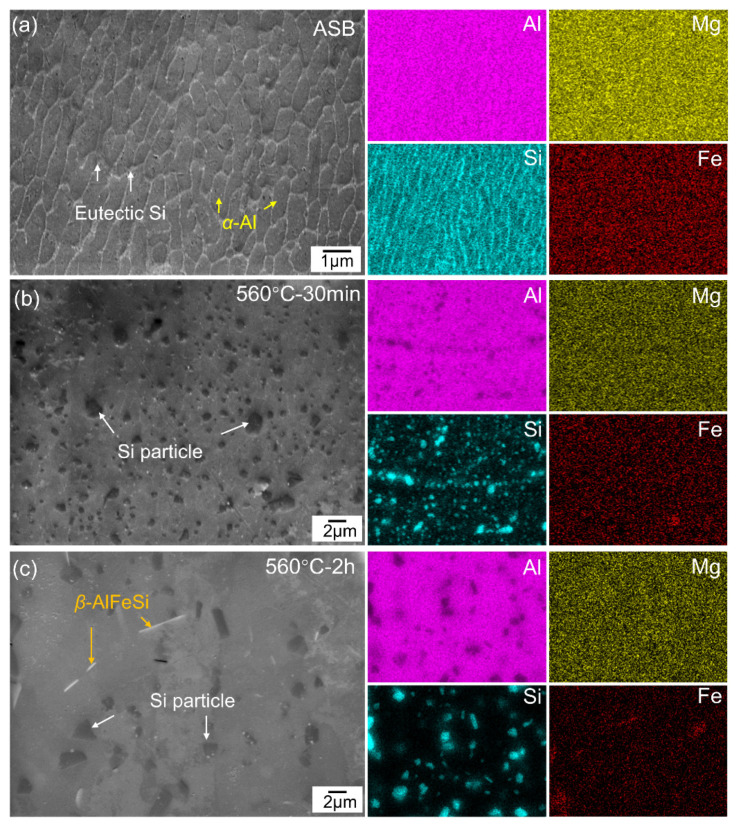
SEM-BSE micrographs and EDS mappings of (**a**) as-built (inside the molten pool), solid solutioned samples at 560 °C for 30 min (**b**) and for 2h (**c**).

Figure 3 shows the SEM-BSE micrographs and energy dispersive spectroscopy (EDS) mappings of the as-built and solution-treated LPBF-processed AlSi10Mg alloys. In the as-built sample (Figure 3a), a typical cellular microstructure with an average cell size of 0.63 ± 0.01 μm is observed at the center area of the melting pool. The Al-Si eutectic structure primarily appears at the α-Al grain boundaries, forming a network structure, with no evident precipitates inside the cells. After solid solution treatment at 560 °C for 30 min (Figure 3b), spheroidization of the Al-Si networks occurs, a phenomenon well-documented in additively manufactured AlSi10Mg alloys [26]. Due to the limited holding time, the segregation of Si particles, with a size of 1.3 ± 0.5 μm, remains visible at the melt pool boundaries. In comparison, extending the holding time to 2 h (Figure 3c) results in the coarsening of Si particles (2 ± 1 μm) and a more even distribution within the Al matrix. The increase in Si particle size with extended holding time suggests a supersaturated state of elemental Si within the Al matrix. According to the Ostwald ripening mechanism [31], larger particles might grow at the expense of smaller ones. Additionally, after 2 h of treatment at 560 °C (Figure 3c), the needle-like *β*-AlFeSi phase is detected, consistent with the observations of Zhu et al. [27].

### 3.2. Microstructure and Hardness Evolution during Artificial Aging Treatment

**Figure 4:**
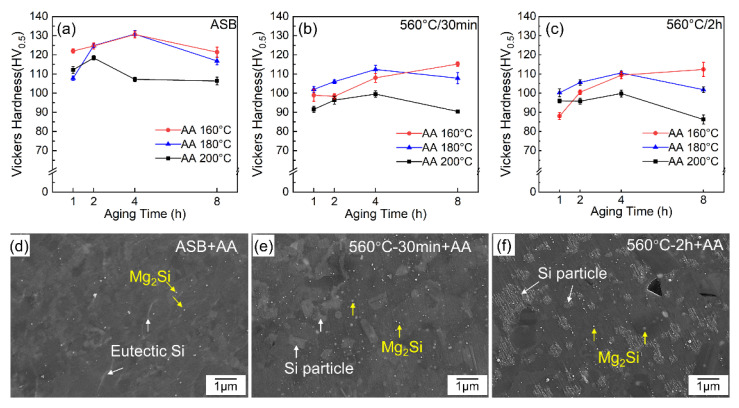
(**a**–**c**) variation of Vickers hardness with the aging time of as-built sample (**a**), solution treated sample at 560 °C for 30 min (**b**) and 2 h (**c**). (**d**–**f**) are representative SEM images of three types of samples aged at 160 °C for 8 h.

To determine the optimal parameters of aging treatment in the present LPBF-processed AlSi10Mg alloy, the as-built and solid solution-treated samples were aged at three temperatures—160 °C, 180 °C, and 200 °C—for durations ranging from 1 to 8 h. The measured Vickers hardness is displayed in Figure 4a–c. Figure 4a illustrates the typical peak aging phenomenon, where the highest hardness was achieved at all three temperatures for the as-built samples (ASB+AA). However, the peak hardness was reached at different holding times: at 4 h for 160 °C (130.6 ± 1.7 HV) and 180 °C (130.8 ± 1.9 HV), while at 200 °C, it was achieved at 2 h. This variation in peak times is likely related to the precipitation dynamics depending on aging temperatures. When the aging time exceeds 2 h, as-built samples aged at 160 °C and 180 °C exhibit higher hardness than those aged at 200 °C. The relatively low hardness of the as-built samples at 200 °C is mainly attributed to softening behavior at the higher aging temperature. In comparison, regardless of the aging temperatures, solid solution-treated samples demonstrated reduced hardness compared to the as-built samples (Figure 4b,c). For 560 °C-30 min samples, unlike the samples aged at temperature as low as 160 °C, which showed a general increase in hardness over time, samples aged at 180 °C and 200 °C exhibit increased hardness values with aging time, reaching their peaks at 4 h. Samples aged at 200 °C demonstrated the lowest hardness values at all aging time points compared to those aged at 160 °C and 180 °C. Similarly, 560 °C-2 h treated samples (560 °C-2 h+AA) showed a comparable trend in hardness variation with 560 °C-30 min+AA samples after aging treatment (Figure 4c). Artificial aging has little influence on the melt pool boundaries, cellular Al-Si networks, and micropores but leads to the precipitation of a high density of Mg_2_Si type precipitates in the matrix [39]. The representative SEM-BSE microstructures after aging treatment at 160 °C for 8 h are shown in Figure 4d–f. Fine Mg_2_Si precipitates are dispersed in the matrix, and with the increase of solution time, the number of these precipitated phases increased significantly.

**Figure 5:**
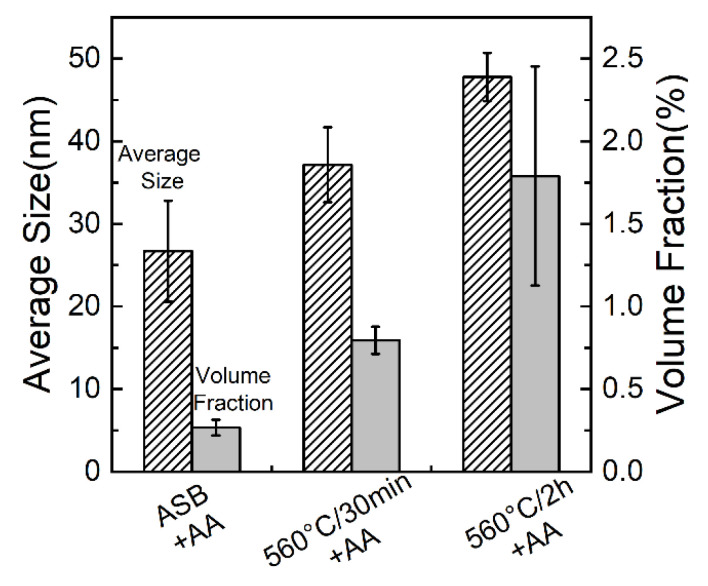
Volume fraction and average size of nano-precipitates in samples after aging treatment at 160 °C for 8 h. Prior to aging treatment, examined samples were treated by as-built state, solid solution at 560 °C for 30 min and 2 h, respectively.

Figure 5 shows a statistical analysis of the sizes and volume fractions of precipitated particles in aged samples with varied solid solution times. The results indicate that the proportion of the precipitated phase in the as-built sample is 0.2 ± 0.04% after directly aging treatment (ASB+AA), while the amount of precipitated phase in the samples pre-treated with solid solution at 560 °C for 30 min (560 °C-30 min+AA) and 2 h (560 °C-2 h + AA) increased to 0.7 ± 0.08% and 1.7 ± 0.6%, respectively. Additionally, the measured sizes of precipitated particles gradually increased with the solution treatment time, ranging from 20 ± 6 nm for the ASB+AA sample to 40 ± 2 nm for the 560 °C/2 h+AA sample. The increased size and volume fraction of precipitates with solid solution time suggests the Si supersaturation within the α-Al matrix.

### 3.3. Tensile Properties and In-Situ XCT Analysis

**Figure 6:**
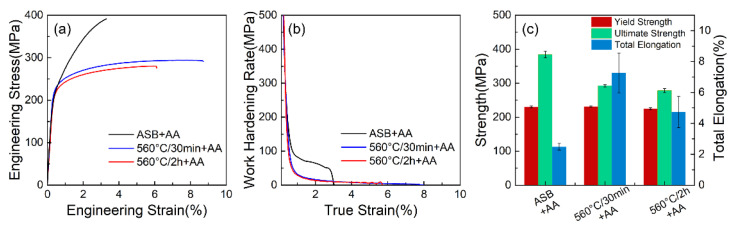
(**a**) engineering stress-strain curves; (**b**) work hardening rate curves and (**c**) tensile property metrics of the LPBF-processed AlSi10Mg alloys after different heat treatments of as-built + artificial aging (ASB+AA), solution treatment at 560 °C for 30 min (560 °C/30 min+AA) and 2 h (560 °C/2 h+AA) followed by artificial aging. Artificial aging (AA) treatments were conducted at 160 °C for 8 h.

To investigate the influence of microstructural changes during solid solution treatment on the tensile properties in the presence of abundant precipitates, an aging treatment at 160 °C for 8 h was employed. This specific protocol was chosen because it resulted in similar high hardness across the three sample types (ASB+AA, 560 °C-30 min+AA, and 560 °C-2 h+AA), as shown in Figure 5a–c. Figure 6a displays the engineering tensile stress-strain curves of LPBF-processed AlSi10Mg alloy after aging treatment. It can be seen that directly aged samples (ASB+AA) exhibit a significantly higher work hardening rate compared to solid solution-treated samples (560 °C-30 min/2 h+AA), as shown in Figure 6b. This results in an enhanced ultimate tensile strength (UTS) of 391.4 ± 1.9 MPa for the ASB+AA samples, while the UTS values are 293.8 ± 1.4 MPa and 280.2 ± 1.3 MPa for the 560 °C-30 min+AA and 560 °C-2 h+AA samples, respectively (Figure 6c). The increased work hardening behaviour observed in the directly aged samples is likely attributed to the synergistic effect of the eutectic Al-Si network (Figure 3a) and nano-precipitates within the grains (Figure 4d). The eutectic Al-Si network at the grain boundary refines the α-Al matrix and acts as obstacles for dislocation movement during tensile tests, significantly enhancing the work hardening rate [40]. However, the directly aged samples show the smallest total elongation (TE, 2.5 ± 0.2%) compared to the solid solution-treated samples (4.9–7.3). The improvement in TE due to solid solution treatment is primarily attributed to the breaking of the eutectic Al-Si network into dispersed Si particles (Figure 3). Compared between two solid solution treated samples, 560 °C-30 min+AA samples demonstrated both higher UTS and TE (Figure 6c). This is conflict with observations by Zhu et al. in LPBF-AlSi10Mg alloys [26]. The reduced total elongation (TE) with extended solid solution time (560 °C-2 h+AA) suggests that porosity plays a significant role in fracture (Figure 2a–c). Additionally, it is interesting to note that although the 560 °C-2 h+AA sample exhibits a higher number density of precipitates (Figure 5) than that of the 560 °C-30 min+AA sample, both aged samples display similar yield strength. This might be due to the different extents of precipitation hardening and solid solution strengthening of the supersaturated matrix.

**Figure 7:**
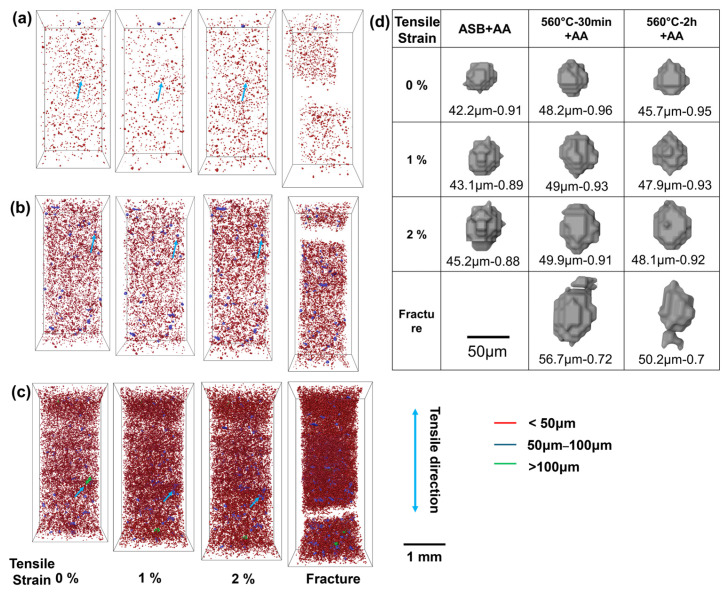
Interrupted in-situ tensile tests with XCT on LPBF-processed AlSi10Mg alloys after heat treatments: (**a**) direct aging treatment at 160 °C for 8 h (ASB+AA), (**b**) solution treatment at 560 °C for 30 min + artificial aging at 160 °C for 8 h (560 °C/30 min+AA), and (**c**) solution treatment at 560 °C for 2 h + artificial aging at 160 °C for 8 h (560 °C/2 h+AA). For each condition, images were captured at four different strains: 0%, 1%, 2%, and at fracture. (**d**) shows the evolution of representative micropores with strain. The equivalent diameter and sphericity of the micropores are also included.

Figure 7 shows the in-situ XCT scanned images of LPBF-processed AlSi10Mg alloy. The XCT scans were performed at four different strains: 0%, 1%, 2%, and at the fracture stage, to reveal the evolution of micropores during interrupted tensile testing. Observations at 0% strain (prior to tensile testing) confirm the significant impact of solid solution time on the size and number density of micropores. The directly aged sample (ASB+AA) primarily comprised micropores smaller than 50 μm, with only a small proportion exceeding this size. As the solution treatment time increased from 30 min to 2 h, both the size and number density of pores increased significantly, particularly for those larger than 100 μm, aligning with the microstructural observations shown in Figure 2a–c. With increasing applied strains (Figure 7a–c), the density and size of micropores increased significantly, especially in the two solid solution-treated samples (560 °C-30 min/2 h+AA), suggesting their dominant role in failure via void growth and coalescence during deformation. To quantitatively describe the evolution of micropores during tensile tests, a representative large pore was monitored under applied strains for each condition, as shown in Figure 7d. To ensure that micropores experience as much as high strain, micropores located near the fracture were typically selected. For all three types of samples, the selected micropores elongated with the applied strains, evidenced by the increased equivalent diameter and decreased sphericity. Additionally, small micropores were observed to attach to the selected large pores at the fracture stage in the two solid solution-treated samples (560 °C-30 min/2 h+AA), indicating the coalescence of micropores at large applied stains. This provides direct evidence of pre-existing micropores influencing the fracture behavior of solid solution treated samples. This is consistent with the observations by Hastie et al. [31] in LPBF-processed AlSi10Mg alloys using XµCT.

### 3.4. Facture Analysis

**Figure 8:**
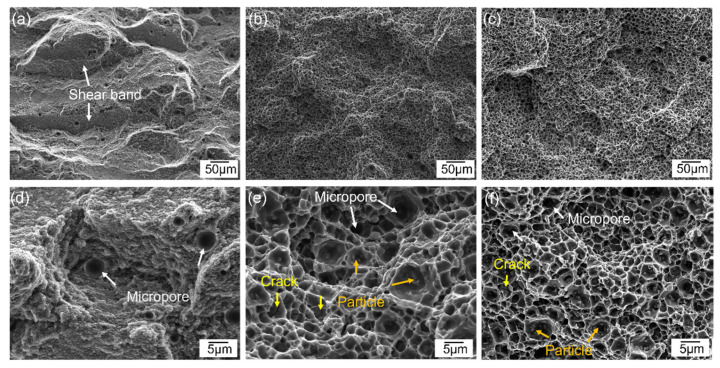
SEM micrographs of fractured surfaces of LPBF-processed AlSi10Mg after different heat treatments: (**a**,**d**) directly aged at 160 °C for 8 h, (**b**,**e**) solid solution treatment at 560 °C for 30 min followed by artificial aging at 160 °C for 8 h, and (**c**,**f**) solution treatment at 560 °C for 2 h followed by artificial aging at 160 °C for 8 h. Images (**d**,**e**,**f**) are enlarged views of (**a**,**b**,**c**), respectively.

Figure 8 shows the fracture surfaces of LPBF-processed AlSi10Mg alloys after different heat treatments. It is evident that although some dimples like features are visible, step-like cleavage planes and shear bands predominate in directly aged samples (ASB+AA), indicating a brittle fracture (Figure 8a,b). This explains the significantly reduced ductility observed during tensile tests, as shown in Figure 6c. In contrast, samples subjected to solid solution treatment exhibit ductile fracture characterized by the presence of predominant dimples (mixed with micropores), as shown in Figure 8e,f. These findings align with their enhanced ductility (Figure 6c). Compared to the 560 °C-30 min+AA samples, the 560 °C-2 h+AA samples demonstrate a more uniform open hole structure (Figure 8e,f). These structures observed in solid solution-treated samples mainly originate from three sources: (1) Si particles, (2) Mg_2_Si precipitates, and (3) micropores. Dimples induced by Si particles and Mg_2_Si precipitates can be distinguished by the presence of faceted particles inside the dimples. Additionally, micro-cracks can be observed at the bottom of many dimples, likely attributed to the Si particles.

**Figure 9:**
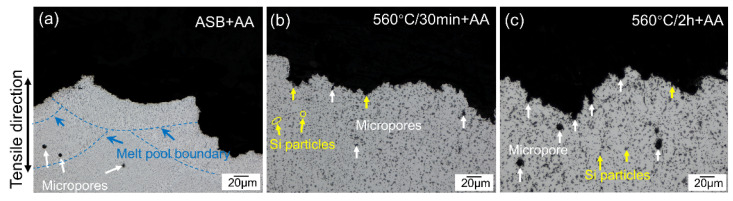
Cross-sectional micrographs of the fractured ends of LPBF-processed AlSi10Mg alloy after different heat treatment: (**a**) direct aged at 160 °C for 8 h (ASB+AA); (**b**) solution treatment at 560 °C for 30 min followed by artificial aging at 160 °C for 8 h (560 °C/30 min+AA) and (**c**) solution treatment at 560 °C for 2 h followed by artificial aging at 160 °C for 8 h (560 °C/2 h+AA).

To further analyze the fracture behavior of the examined samples, the cross-sectional microstructures at the fractured ends were characterized, as displayed in Figure 9. In Figure 9a, cracks tend to propagate along the melt pool boundaries in the directly aged samples (ASB+AA), where the Al-Si network is prominent (Figure 2a). This indicates that the fracture of ASB+AA samples is primarily influenced by the inherent microstructural features of printed AlSi10Mg. With the dissolution of the melt pool boundaries during solid solution treatment, the crack propagation pattern in 560 °C-30 min+AA samples shifted to follow the boundaries of Si particles and micropores, as detailed in Figure 9b. As the solid solution time was extended to 2 h (560 °C-2 h+AA), the influence of micropores on crack propagation became more significant due to their growth (Figure 9c), resulting in a reduced ductility (Figure 6c).

## 4. Conclusions

This study systematically investigated the effect of T6 heat treatment (solid solution plus aging treatment) on the microstructure, micropore evolution, and mechanical properties of LPBF-processed AlSi10Mg alloy. The main findings are as follows:

(1) Solution treatment at 560 °C results in the dissipation of eutectic Al-Si networks into isolated Si particles. Extending the solid solution treatment at 560 °C significantly increases the size and number density of micropores.

(2) The samples directly aged at 160 °C for 8 h exhibit a remarkably high work hardening rate, contributing to the highest ultimate tensile strength (UTS) of 391.4 ± 1.9 MPa, but with a total elongation (TE) of only ~2.6%. In contrast, T6 treatment significantly enhances ductility due to the granulation of eutectic Al-Si networks, particularly at the melt pool boundaries. Compared to samples treated at 560 °C for 2 h (560 °C-2 h+AA), samples treated for a shorter period (560 °C-30 min+AA) exhibit both higher UTS and TE.

(3) Interrupted in-situ tensile testing combined with XCT reveals that large micropores elongate in the loading direction. For the two T6-treated samples (560 °C-30 min/2 h+AA), evident coalescence of micropores is observed at the fracture stage.

(4) The directly aged samples (ASB+AA) exhibit brittle fracture characteristics, with cracks occurring primarily along the melt pool boundary where eutectic Al-Si networks are predominant. In contrast, after T6 treatment, crack propagation shifts to dominated by Si particles and micropores. As the solid solution time extends in the T6 treatment, the influence of micropores on crack propagation becomes more significant.

## Data Availability

The original contributions presented in the study are included in the article, further inquiries can be directed to the corresponding author/s.
